# Oxidative Stress, Genomic Integrity, and Liver Diseases

**DOI:** 10.3390/molecules27103159

**Published:** 2022-05-15

**Authors:** Nanthini Sadasivam, Yu-Ji Kim, Kamalakannan Radhakrishnan, Don-Kyu Kim

**Affiliations:** 1 Department of Integrative Food, Bioscience and Biotechnology, Chonnam National University, Gwangju 61186, Korea; 218545@jnu.ac.kr (N.S.); 178252@jnu.ac.kr (Y.-J.K.); 2 Clinical Vaccine R&D Center, Department of Microbiology, Combinatorial Tumor Immunotherapy MRC, Medical School, Chonnam National University, Gwangju 58128, Korea

**Keywords:** oxidative stress, genomic integrity, gene regulation, liver disease

## Abstract

Excess reactive oxygen species production and free radical formation can lead to oxidative stress that can damage cells, tissues, and organs. Cellular oxidative stress is defined as the imbalance between ROS production and antioxidants. This imbalance can lead to malfunction or structure modification of major cellular molecules such as lipids, proteins, and DNAs. During oxidative stress conditions, DNA and protein structure modifications can lead to various diseases. Various antioxidant-specific gene expression and signal transduction pathways are activated during oxidative stress to maintain homeostasis and to protect organs from oxidative injury and damage. The liver is more vulnerable to oxidative conditions than other organs. Antioxidants, antioxidant-specific enzymes, and the regulation of the antioxidant responsive element (ARE) genes can act against chronic oxidative stress in the liver. ARE-mediated genes can act as the target site for averting/preventing liver diseases caused by oxidative stress. Identification of these ARE genes as markers will enable the early detection of liver diseases caused by oxidative conditions and help develop new therapeutic interventions. This literature review is focused on antioxidant-specific gene expression upon oxidative stress, the factors responsible for hepatic oxidative stress, liver response to redox signaling, oxidative stress and redox signaling in various liver diseases, and future aspects.

## 1. Introduction

### Oxidative Stress and Antioxidant Response

Oxidative stress is caused by an imbalance between reactive oxygen species (ROS) and antioxidants. ROS are mainly oxygen-based hydroxyl group molecules. Along with reactive nitrogen species (RNS, nitrogen-based anion groups), ROS production takes place in a natural cellular process. To maintain cell integrity, ROS levels should be adequately balanced with antioxidant defenses [1,2,3]. The balance between free radicals and antioxidants is a well-controlled process in cells to escape oxidative stress and cell damage [4]. A steady level of ROS is maintained by ROS clearance pathways [5] and physiological redox signaling is stimulated in response to ROS [6]. In physiological functions like cell metabolism, cell survival, epigenetic pathways, immune defense, and transcription factor modulation, a normal level of ROS is maintained to act as signaling molecules [7,8]. Under a pathological condition, excess ROS can result in the stimulation of pathological redox signaling that can cause cellular damage and various disease conditions [9]. Due to the oxidative stress produced during pathological and physiological conditions under aerobic metabolism, various organic compounds such as DNAs, lipids, carbohydrates, and proteins are structurally damaged. A proper oxidant level is maintained by a natural system called the antioxidant defense system that acts by both enzymatic and non-enzymatic processes [10,11].

The process of oxidant formation begins by oxygen being reduced to water and, as a result, it produces free radicals such as superoxide anion radical (O_2_^−^), hydrogen peroxide (H_2_O_2_), and hydroxyl radicals. Mitochondrial complexes, NADPH oxidases, peroxisomes, microsomal electron transport chain (ETC), oxidase, cyclooxygenases, and xanthine oxidase are the biological machinery involved in the oxidant formation [5,12]. The mitochondria play a major role in formation of oxidants, as mitochondrial complex-I and II in the ETC pass the electrons from reduced substrates through other mitochondrial complex-III and IV to oxygen, and thus forming water and resulting in O_2_^−^ formation. Complex-I in the mitochondria produces the highest amount of superoxide. The oxidation of succinate is the major process in complex-I, which is then used as a substrate, succinate dehydrogenase, for complex-II [13]. The major enzyme involved in the production of free radicals is NADPH oxidase, a multicomponent enzyme complex containing two membrane and three cytosolic components [14,15]. This enzyme converts the oxygen to O_2_^−^ by the reaction of NADPH along with oxygen to a reduced form of NADP^+^ and O_2_^−^, while further biochemical process reduce superoxide to H_2_O_2_ [16,17]. Peroxisomes are the other major source of ROS in the form of H_2_O_2_, produced by the peroxisomal enzymes like catalase (CAT) and glutathione peroxidase (GPx) [18]. The half-life of ROS depends on the type of defense system or mechanism (i.e., different oxidants will participate in the reaction depending on the target molecule and the site of generation). One such example is peroxyl radicals, an oxidant species in cell metabolism, which is transported to a distant target site to exhibit oxidant activity [19].

Cells have antioxidants, the substances that play a major role in inhibiting the oxidation of an oxidizable substrate, naturally to balance levels of oxidants in the system [20]. The protection by antioxidants is achieved by converting ROS to less harmful products through a cascade of reactions involving both nonenzymatic and enzymatic compounds [21]. The antioxidant nonenzymatic compounds such as α-tocopherol (vitamin E), ascorbic acid (vitamin C), flavonoids, β-carotene (vitamin A), and many other compounds can prevent damage caused by ROS [22]. Antioxidant enzymes such as CAT and superoxide dismutase (SOD) act against H_2_O_2_; however, CAT and SOD enzymes are not very effective in limiting the interaction of ROS with macromolecules that are highly reactive to oxidative stress. Thus, a second line of defense enzymes, such as GPx and glutathione-S-transferase (GST), aldo-keto reductase and aldehyde dehydrogenase (ALDH), are essential against ROS. Glutathione, a molecular compound in both reduced (GSH) and oxidized (GSSG) form, is involved by acting as a coenzyme for various enzymes and also as a substrate for GPx and GST during the cell defense mechanism against lipid and hydrogen peroxidation [23,24,25]. It is important to eliminate the secondary metabolites formed during this enzyme activity, and it is achieved by the glutathione S-transporter, thus preventing the cellular damage and cell apoptosis [24]. These naturally occurring antioxidants can offer defense against the damaging effects of ROS. Besides natural antioxidants, synthetic antioxidants in the form of drugs are also used to provide defense against reactive oxygen metabolites [10].

## 2. Factors Responsible for Oxidative Stress in the Liver

### 2.1. Alcohol-Induced Oxidative Stress

Alcohol is a widely used beverage; however, higher doses and binge drinking of alcohol consumption can cause serious health problems, including liver diseases ranging from very mild to fatal end stage liver disease. Alcoholic liver disease (ALD) is associated with excessive alcohol consumption, resulting in liver damage [26]. Alcohol metabolism involves two main steps. First, alcohol dehydrogenase (ADH) converts alcohol to acetaldehyde (toxic reactive molecule), followed by the action of ALDH to convert acetaldehyde to acetate. During alcohol metabolism, a molecule of nicotinamide adenine dinucleotide in a reduced form hydrogen (NADH) is formed and, as a result of oxygen consumption, ROS production is increased [23]. ROS production based on alcohol consumption involves other sources, such as NADH-dependent cytochrome reductase, aldehyde oxidase, xanthine oxidase, and neutrophil nicotinamide adenine dinucleotide phosphate. The generation of O_2_^−^ and H_2_O_2_ is associated with the cellular source of ROS production and the precursor of O_2_^−^ [27]. Hepatitis, fibrosis, and cirrhosis are severe forms of liver diseases that potentially lead to fatal liver failure [28]. Alcohol metabolism is directly associated with ROS production and mitochondrial injury that can be caused by acute and chronic exposure to alcohol [29]. Studies using animals exposed to alcohol have shown decreased effects due to both enzymatic and nonenzymatic systems by which cellular homeostasis is maintained [30]. The enzyme activities of SOD, CAT, GPx, glutathione reductase, and GST are reduced, and lipid peroxidation levels are increased in animals exposed to alcohol [31]. When the oxidative stress and levels of GPx were measured, it was found that serum levels of the lipid peroxidation indicator malondialdehyde (MDA) were elevated after exposure to alcohol. The elevated level of MDA affects the antioxidant activity and is also associated with the serum CAT, since CAT is important in eliminating H_2_O_2_ [32]. Vitamin-E and vitamin C, as indicators of the antioxidant system, were decreased in patients with ALD [33]. Studies have shown that levels of oxidative stress are increased in individuals suffering from ALD [34].

### 2.2. Drug-Induced Oxidative Stress

The liver is an important organ that performs various vital biological functions, including drug metabolism. Hepatic drug metabolism is a complex process involving enzymes at two phases. The first phase involves oxidation by cytochrome p450. The second phase involves reduction and hydrolysis, followed by the conjugation or coupling with molecules (i.e., glucuronidation, sulfonation), making the catabolite polar to be excreted into the bile [35]. Hepatic accumulation of drugs or its by-products can cause serious liver injury and dysfunction. Drugs are metabolized by the liver to form water-soluble compounds as end products which are then expelled to the bile. Previous studies and discoveries have proclaimed that a lot of drugs are responsible for oxidative stress induction. Anti-inflammatory drugs, anticancer drugs, antianalgesic drugs, and antidepressants can all increase cellular oxidants and lipid peroxidation and lead to the depletion of antioxidants in the liver [36]. Extensive use of analgesics has been tested using mice. Results showed that MDA, a marker of oxidative stress and nitrate in the liver, was increased, whereas the activities of SOD and Cu-Zn/SOD were decreased [37]. The connection between drugs and hepatic oxidative stress has been studied after a chronic administration of fluoxetine and clozapine. Results showed increased MDA and alanine transaminase (ALT) activities but decreased GSH levels [38]. Anticancer drugs such as cisplatin, doxorubicin, docetaxel, and paclitaxel can induce oxidative stress in the liver, either directly and/or indirectly, through DNA damage [39,40,41]. For example, doxorubicin and paclitaxel can increase thiobarbituric acid-reactive substance and docetaxel can reduce SOD activity [39]. Anti-inflammatory drugs, such as nimesulide, and nonsteroids can increase the activities of ALT, alkaline phosphatase, and aspartate aminotransferase (AST), but decrease the activity of SOD [42]. Evidence on the hepatic damage in patients due to drug-induced oxidative stress is insufficient. Human cells have been mimicked to study the pathogenesis of hepatotoxicity induced by drugs in humans; nevertheless, antioxidant treatment of hepatotoxicity induced by drugs requires sufficient data and information through future studies [43].

### 2.3. Other Factors Inducing Oxidative Stress

Radiation, environmental agents, and temperature are other factors that can induce oxidative stress. Studies have been conducted to explore the role of radiation and its link to oxidative stress [44,45]. When the liver of a guinea pig is exposed to mobile phone-like radiofrequency radiation, it has been found that MDA and nitric oxide are increased whereas the level of SOD is decreased [46]. Studies have shown that cold stress can also induce oxidative stress [47,48]. Benzoyl peroxide is a bleaching agent that can induce oxidative stress which results in reduced antioxidant levels and antioxidant enzyme activity [49]. Nanoparticle exposure can affect the viability of hepatic cells [50], and, as a result, it can decrease levels of enzymes related to oxidative stress. Other disease conditions include factors affecting liver damage caused by oxidative stress. Several markers and antioxidant enzymes in autoimmune disorders have been studied and analyzed [51,52]. Many studies have investigated the link between oxidative stress and other diseases; however, such a link has not been fully defined yet. Other factors such as environmental pollutants can also induce oxidative stress. Environmental pollutants such as heavy metals like mercury have been studied in mice and rats [53]. Lead, as another pollutant, can aggravate liver lipid peroxidation by which free radicals play a role in the pathological mechanism of lead-induced hepatotoxicity. Algae toxin microcystin possesses liver toxicity that can damage the liver and reduce antioxidant enzyme levels [54,55,56].

## 3. Hepatic Oxidative Stress Response and Genomic Integrity of Redox Signaling

Redox signaling, a conserved mechanism in organisms ranging from bacteria to higher eukaryotes, is totally controlled by the signal transduction pathways [57]. ROS conditions can affect the signal transduction pathway by influencing cellular gene expression. Gene expression patterns can be modified in response to stress by activating the redox sensitive transcription factor and the antioxidant responsive element (ARE) genes [58]. The oxidant-dependent cellular signaling in the liver needs a better understanding, and further studies are needed to develop specific therapeutics for liver injuries and diseases.

### 3.1. Transcriptional Regulation and Redox Homeostasis

The liver is an organ which is excessively accumulated with fats, metabolic products, and toxins which disturb the redox state and damage the cells. To maintain the redox state in the liver, antioxidants play a major role in treating liver damage during the ROS condition caused by various enzymes, as discussed earlier. Activation of the genes encoding these enzymes is controlled by *cis*-acting regulatory elements, which are called AREs, in the promoter gene. As illustrated in Table 1, several antioxidant genes are regulated in the liver in response to oxidative stress. ARE genes include, but are not limited to, GST, NAD(P)H: quinone oxidoreductase, and the glutamate-cysteine ligase catalytic subunit. ARE gene expression is regulated by the leucine zipper transcription factor called nuclear factor erythroid 2-related factor 2 (Nrf2), which regulates cellular antioxidant response genes [59]. Electrophiles and radicals-activated Nrf2 disassociates from kelch-like ECH-associated protein 1 (Keap1) and translocate into the nucleus to activate ARE-responsive genes. Nrf2 protects the liver from toxins that can lead to acute and chronic disease conditions, and hepatic Nrf2 deficiency has showed enhanced oxidative stress [59]. Nrf2 also positively regulates liver regeneration through the regulation of the expression of the augmenter of liver regeneration (ALR), a liver survival factor with antiapoptotic and proliferative efficiency [60]. Another gene regulated in the liver in response to oxidative stress is estrogen related receptor γ (ERRγ). ERRγ, along with ERRα and ERRβ, represents the NR3B subfamily of nuclear receptors [61]. ERRγ is mainly involved in liver metabolic diseases such as alcohol-induced oxidative stress, type 2 diabetes, liver injury, dysfunction of insulin signaling, iron metabolism, and microbial infections by liver gluconeogenesis [62,63,64]. ERRγ exhibits a similar structure to nuclear receptors with a poorly conserved N-terminal domain of activation function 1 (AF-1), a central DNA-binding domain, and a C-terminal ligand-binding domain with an AF-2 domain. Interactions of AF-2 with coactivators or with corepressors regulate the transcriptional activity of ERRγ [64,65]. Studies have reported that ERRγ regulation is linked to liver glucose metabolism, as ERRγ is directly associated with gluconeogenesis by regulating the expression of key gluconeogenic genes in the liver [62,66,67]. ERRγ function is not restricted to glucose metabolism, as it is also involved in alcohol and lipid metabolism [68,69]. The most important enzyme in alcohol metabolism is cytochrome P450 2E1 (CYP2E1), which can cause alcohol-induced ROS and result in liver injury called ALD. The CYP2E1 gene is regulated by ERRγ, an active transcriptional activator regulating gene expression [70]. CYP2E1 is involved in metabolizing compounds such as alcohol, benzene, halothane, and halogenated substrates [71,72,73]. Experiments conducted with ethanol-treated rats have shown that the increase in ROS production and lipid peroxidation can be blocked by CYP2E1 chemical inhibitors and anti-CYP2E1 immunoglobulin G [74]. Increased NADPH oxidase activity has been found for CYP2E1 because of a defective interaction between NADPH and cytochrome P450 reductase [75]. Earlier studies have experimentally shown that CYP2E1 is not involved in alcohol-induced liver injury [76,77]; however, further information on this is needed. Recent research suggests that ERRγ gene expression in alcohol-induced oxidative stress is induced by the CB_1_ receptor, which induces CYP2E1 [70]. ERRγ controls the regulation and pathogenesis of alcohol-induced oxidative stress and liver injury by CYP2E1 induction [70]. Mechanisms for alcohol-induced liver injury and oxidative stress mainly involve CYP2E1 and other sources, such as mitochondria and Kupffer cells. ROS generation is caused by CYP2E1, O_2_^−^, and H_2_O_2_ as oxidative products during the metabolic process. Stronger oxidants are produced by the addition of iron, which has been discussed earlier. CYP2E1-associated oxidative liver injury in liver cells can respond to oxidative stress by transcriptionally active antioxidant enzymes through antioxidant repair or response pathways and products [78]. The regulation of genes in the liver other than carbohydrate and lipid metabolism includes iron metabolism, which also plays a significant role in liver disease. It is important to address genes regulated during iron metabolism causing liver diseases. Epigallocatechin-3-gallate (EGCG) can induce small heterodimer partner-interacting leucine zipper protein (SMILE) expression by forkhead box protein 1 [79]. Studies have shown that SMILE is a transcriptional corepressor of NR, which controls the glucose and lipid metabolism [80,81,82,83]. SMILE can inhibit the action of interleukin-6-mediated signal transducer and activator of transcription 3 (STAT3) on the iron metabolism. Upregulation of SMILE can suppress Janus kinase-2 signal transducer involved in hepcidin synthesis by inflammatory factor IL-6. STAT3 can promote the production of hepcidin, a key regulator factor in iron metabolism. EGCG can induce SMILE and inhibit STAT3 to bind to the hepcidin gene promoter [79]. SMILE expression also plays a role in the endoplasmic reticulum (ER) stress response [84]. Curcumin, a natural antioxidant, is used as a treatment for the ER stress response. Liver-enriched cyclic AMP responsive element-binding protein H (CREBH) and ATF-6 are ER-bound transcription factors activated during ER stress. Curcumin can induce SMILE activity by liver kinase B1 and the AMP-activated protein kinase signaling pathway. SMILE upregulation can repress CREBH activity [84].

**Table 1:** List of hepatic oxidative stress responsive gene and its function in preventing liver diseases.

Gene	Function	Liver Disease	Ref.
Glutathione-S-transferase (GST)	- Antioxidant response	- Liver metastases	[85]
		- Alcoholic liver steatosis	[86]
		- Nonalcoholic fatty liver disease	[87]
Glutamate-cysteine ligase catalytic subunit (GCLC)	- Antioxidant response	- Liver fibrosis	[88]
NAD(P)H:quinone oxidoreductase 1 (NQO1)	- Antioxidant response	- Nonalcoholic fatty liver disease	[89]
		- Drug-induced liver injury	[90]
Nuclear factor erythroid 2-related factor (Nrf2)	- Antioxidant response	- Drug-, chemical-, or virus-induced hepatitis	[59,91]
		- Alcoholic or nonalcoholic steatohepatitis	[92,93]
		- Fibrosis and cirrhosis	[94,95]
		- Hepatocellular carcinoma	[96,97]
		- Hepatic ischemia–reperfusion injury	[98,99]
Estrogen-related receptor γ (ERRγ)	- Oxidative stress response	- Alcoholic fatty liver	[100]
		- Alcoholic liver injury	[70]
		- Hepatocellular carcinoma	[101]
Cytochrome P450 2E1 (CYP2E1)	- Oxidative stress response	- Nonalcoholic steatohepatitis	[102]
		- Halothane hepatitis	[103,104]
Small heterodimer partner-interacting leucine zipper protein (SMILE)	- ER stress response	- Iron deficiency in liver	[84]
Heme oxygenase-1 (HO-1)	- Antioxidant response- Oxidative stress response and redox homeostasis maintenance	- Chronic hepatitis	[105,106]
		- Hepatic ischemia–reperfusion injury	[98,107,108]
		- Nonalcoholic fatty liver disease	[87]
Apurinic-apyrimidinic endonuclease/Redox effector factor-1 (APE-1/Ref-1)	- Activation of transcriptional factor in response to ROS and redox homeostasis maintenance	- Hepatocellular carcinoma	[109]

### 3.2. Enzymatic Regulation and Redox Homeostasis

Heme oxygenase (HO) is a key enzyme which breaks down heme into carbon monoxide (CO), iron, and bilirubin. HO has three isoforms, namely HO-1, HO-2, and HO-3. HO-1 is the most actively expressed isoform of HO. HO-2 and HO-3 are expressed along with HO-1 expressions. By-products such as CO and biliverdin/bilirubin have been shown to have protective effects against oxidative stress causing liver damage [110]. Under extreme oxidative conditions or hypoxia/hyperthermia, HO-1 is induced to catabolize heme in higher amounts [111,112]. The HO pathway begins by degrading heme and converting it into biliverdin, CO, and iron. Biliverdin is converted into bilirubin by an enzyme called biliverdin reductase, and bilirubin functions as an anti-inflammatory, antioxidant, and antiapoptotic, which helps in maintaining redox homeostasis. HO-1 is an important regulatory factor in liver protection [110]. Various stressors, inducing factors such as UV, hyperoxia, lipopolysaccharide, and heme-induced damage, can affect the liver, cause oxidative stress, and induce HO-1 [113,114]. Several studies have proven that HO-1 expression upregulation against various diseases is caused by oxidative stress [115]. Studies using a systemic model of rat hemorrhagic shock and revival, male mice with ischemia and reperfusion, and leukocyte accumulation in the liver have demonstrated the role of HO-1 expression in hepatoprotection [110,116]. HO-1-deficient mice were prone to hepatic necrosis, anemia, growth retardation, and iron deposition in the liver and other organs [114]. Transcription function and induction of HO gene expression are regulated by several signaling molecules, including protein kinases as upstream signaling molecules and transcription factors as downstream molecules. Extracellular signal-regulated protein kinases (ERKs), c-Jun N-terminal kinase (JNK), and p38 MAP kinase have a major role in HO expression and regulation [117]. Other enzymes, such as protein kinases, protein kinase A (PKA), and phosphatidylinositol-3 kinase (PI3K), are also responsible for upregulating HO expression. Protein kinase-associated factors are also involved in repairing liver damage. PKA can be induced by intracellular cAMP due to external and internal stimuli. Studies using rat hepatocytes treated with PKA, inducing factor Bt_2_cAMP and glucagon, have shown HO-1 upregulation [112,118]. Studies have also demonstrated that protein kinase-associated factors play a vital role in HO expression, as PKA and PI3K/AKT pathways can also control ROS levels and regulate the expression of HO. The PI3K/AKT pathway is also involved in the post-translational activity of HO-1 [119]. The action of protein kinases and molecular expression related to HO-1 expression remain unknown. The second intracellular signaling-regulating factor in HO-1 expression for protecting the liver is the transcription factor. HO-1 expression is regulated by various redox-sensitive transcription factors such as activator protein-1 (AP-1), nuclear factor kappa-light-chain-enhancer of activated B cells (NF-κB), and Nrf2. Upon external stress/stimuli, transcription factors in the cytosol can bind to specific promoter regions/genes (AREs) in the DNA. Transcription factors can be translocated to the nucleus to induce HO-1 expression upon binding, resulting in liver protection [110,112].

### 3.3. Genomic Integrity and Oxidative Stress

**Figure 1:**
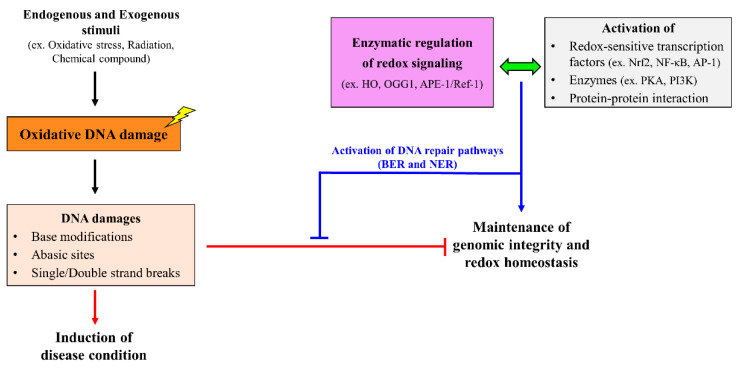
Enzymatic regulation of redox homeostasis and genomic integrity.

Redox regulations are associated with various factors like proteins, transcription factors, gene expressions, and several cellular pathways. ROS and RNS, along with many other factors, induce DNA damage and thereby disturbs the integrity of genome [120]. Radiations, oxidative stress, chemical compounds, and other external and internal factors are also responsible for DNA damage [121,122]. Oxidative stress can induce specific base modifications in a DNA, such as 7, 8-dihydro-8-oxodeoxyguanosine (8-OXO-dG)/GC or TA transversion, because of a high reactivity with a strong nucleophilic action [123]. Upon DNA damage, DNA damage response pathways are activated to repair the damaged DNA in order to maintain the genome integrity [124]. Different repair mechanisms are involved in the repair of DNA lesions, damage, and modification. Base excision repair (BER) is activated during base modifications in the DNA. For BER, the BER enzymes are activated during the repair mechanism [125]. Detailed information on the BER pathway is discussed below. Nucleotide excision repair (NER) is important for repairing complex DNA damage [126]. Various transcription factors, including redox-sensitive transcription factors such as NF-κB, Nrf2, estrogen receptor, hypoxia-inducible factor-1α (HIF-1α), and transcription cofactor RNA helicase are involved in DNA damage. Transcription factors can bind to DNA motifs or other transcription factors, such as AP-1, specificity protein-1, paired-like homeodomain-1, and runt-related transcription factor 1, through protein–protein interactions [127,128,129]. Each transcription factor exhibits different roles. Nrf2 is activated upon thiol oxidation. It can regulate protein Keap1 and protect antioxidants [129,130]. ROS/RNS can also affect the mRNA stability by the redox modifications of proteins that bind to AU-rich sites. Oxidative stress and redox signaling have a high impact on DNA repair, genomic stability, and gene regulation (Figure 1). One such enzyme involved in maintaining redox homeostasis is HO.

External and internal stimuli can result in oxidative DNA damage and lead to various diseases. Genomic stability and redox homeostasis can be disturbed due to DNA damage, such as base modifications, the loss of nucleobase (abasic site), and single/double strand breaks. The repair mechanism for oxidative DNA damage is activated by several enzymes, such as HO, OGG1, and APE-1/Ref-1, which are responsible for activating transcription factors and pathways that can repair DNA damage and maintain redox homeostasis and genomic integrity. BER, base excision repair; NER, nucleotide excision repair.

Apurinic-apyrimidinic endonuclease-1 (APE-1) enzyme is another important regulator in cellular response to oxidative stress by the DNA repair function and activating Nrf2 and NF-κB transcription factors. It is involved in the BER pathway against oxidative damage. APE-1 is an enzyme activator for transcription factors to activate AP-1, NF-κB, HIF-1α, paired box gene 8, and so on, in response to ROS [131]. It is important to maintain redox homeostasis by regulating the transcription factors and by activating enzymes (thioredoxin, CAT, and SOD). Apart from regulating and activating transcription factors, the enzymes APE-1/Ref-1 also depend on the BER pathways, stress response, and energy metabolism. The DNA repair function of APE-1/Ref-1 begins in the C-terminal region (active site) with three residual domains forming a loop. The APE-1/Ref-1 enzyme is inserted in the loop. It can flip the AP nucleotide into a hydrophobic end and kink the DNA helix. The nucleotide side is then responsible for the redox activity. Studies have shown that APE-1/Ref-1 also has a role in the post-transcriptional regulation of genes such as HO [132,133,134]. The role of APE-1/Ref-1 in DNA damage repair is vital. Oxidative DNA damage is responsible for various diseases. DNA damage by the oxidative condition can alter gene sequences, disrupt signaling pathways, and form DNA lesions. These DNA lesions can be repaired by the BER pathway [135]. Upon DNA damage caused by oxidative stress, single nucleotide BER (SN-BER) is activated by the monofunctional DNA glycosylases (DGs) enzyme that can alter the AP site via APE-1 and produce 3′-OH group and 5′dRP (blocking group). The blocking group is cleaved by the action of poly β, and the DNA ligase then completes the repair mechanism and restores the genome integrity [136,137,138,139]. Like the SN-BER pathway, in the long patch BER (LP-BER) pathway, the bifunctional enzymes with DG and AP lyase activity can cleave the AP site and allow the binding of 3′ phosphate (blocking group). By the action of APE-1, the blocking group is cleaved from the AP site by the action of poly δ and poly ε, flap endonuclease-1, and proliferating cell nuclear antigen, followed by the ligase 1-mediated DNA repair completion. Thus, the SN-BER and LP-BER pathways play a vital role in maintaining the genomic integrity [140,141,142]. An important BER enzyme possessing bifunctional DG and AP lyase activity is 8-oxoguanine glycosylase 1 (OGG1). It directly identifies the base damage in DNA, and the most frequent base-damaged DNA lesions, 8-OXO-dG, caused as a result of free radical formation, is excised along with imidazole ring-fragmented guanine lesions. The activity of OGG1 begins by flipping the damage base from the DNA double helix and cleaves the N-glycosidic bond of the damaged base, leaving the AP-site, and then executes the lyase activity, and cleaves a single stranded break, followed by recruiting other DNA repair partners [143,144]. A study on WT and OGG1 knockdown mice showed the importance of OGG1, as evidenced by the increased 8-OXO-dG lesions in OGG1 knockdown mice [145]. Decreased OGG1 activity leads to various diseases, including cancer, neurodegenerative diseases, and metabolic conditions, due to oxidatively-induced DNA damage [146].

## 4. Oxidative Stress and Redox Signaling in Liver Diseases

**Figure 2:**
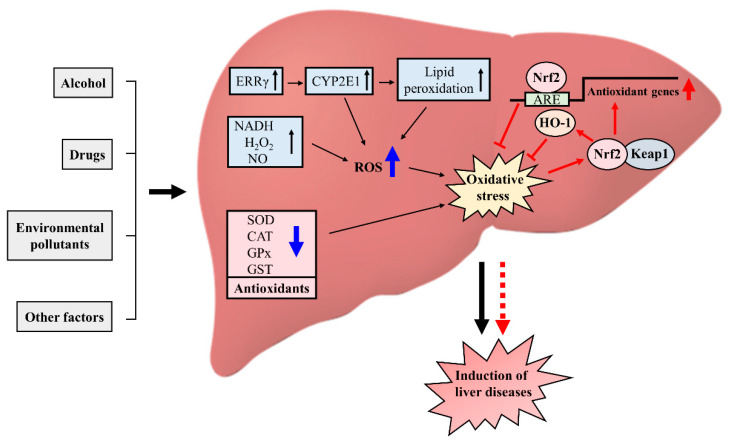
Various factors causing redox imbalance and oxidative stress and induction of liver diseases.

ROS play a very important role in the induction of various diseases, as previous studies and experiments on liver diseases have shown evidence of liver diseases, damage, and injury under an oxidative stress condition [147]. Disease pathogenesis in the liver involves all cell types, including hepatocytes, Kupffer cells, stellate cells, and liver sinusoidal endothelial cells [148,149,150]. Oxidative damage can lead to altered gene expression as a result of apoptosis, necrosis, ischemia, and so on [151]. Cytochrome P450 enzyme, neutrophil, hepatocyte mitochondria, and endotoxin–Kupffer cells produce free radicals [152]. As discussed above, the redox signaling pathways which are involved in redox-sensitive transcription factors and enzymes are important in preventing liver disease due to the oxidative condition (Figure 2). The evidence on molecular targets of oxidative stress in the liver is limited. Molecular markers are needed to develop new therapeutic approaches.

Various factors, such as alcohol, drugs, and other factors, are responsible for oxidative stress and ROS production. These factors can induce the oxidant products like NADH, H_2_O_2_, and NO that are responsible for ROS generation, and can also reduce the activity of antioxidants like SOD, CAT, GPx, and GST. In addition, excessive alcohol consumption can increase the gene transcription of ERRγ and CYP2E1, involved in elevated levels of lipid peroxidation, resulting in oxidative stress in the liver and which can cause liver disease. During the oxidative stress condition, several ARE genes and enzymes are activated, which inhibit the oxidative stress response and control the liver diseases.

### 4.1. Alcoholic Liver Disease

Acute and chronic liver diseases are mainly affected by alcohol metabolism. They can result in the increased production of ROS, oxidative stress, and low antioxidant levels. Ethanol-induced oxidative stress is evident in many studies [153]. ALD has a broad range of disease conditions, including hepatic steatosis, hepatitis, and cirrhosis, that can lead to hepatocellular carcinoma. Mechanisms involved in the oxidative stress response during ALD include redox state change, damage to mitochondria, the ethanol induction of CYP2E1 expression, the ethanol mobilization in iron, and effects on the antioxidant system. Among them, damage to mitochondria is the main cause of oxidative stress. Several proapoptotic factors (such as cytochrome and caspase-3), and other factors such as HIF-1α, can intensify liver injury in response to improper metabolism in the liver [154]. Alcohol liver injury can be prevented by replacing polyunsaturated fat with saturated fat or medium chain triglycerides in the diet of rats [155]. Alcohol liver injury is directly associated with lipid peroxidation. Iron plays an important role in promoting oxidative stress. Adding iron to the diet can generate OH and promote the oxidative stress response by elevating the lipid peroxidation [156]. Less reactive oxidants, such as superoxide and H_2_O_2_, can be converted to high reactive oxidants by a catalyzing process which promotes oxidative stress by iron [157]. Alcohol-induced cirrhosis is a condition where iron uptake is elevated [158]. Ethanol treatment can lead to high iron levels in liver cells [159]. Ethanol is considered important in the oxidative stress response to liver injury in Kupffer cells and hepatocytes, where NF-κB activation is important for tumor necrosis factor-α (TNF-α) production and ALD [160]. CYP2E1 is involved in ROS production and lipid peroxidation in alcohol liver injury [161], and its expression is upregulated after the addition of iron, which causes iron toxicity [162]. Moreover, several functions of mitochondria, such as ATP concentration, blood alcohol level, and oxygen uptake of hepatic cells, are decreased during alcohol induction [163]. Mitochondria dysfunction by ethanol can increase ROS production and toxicity, which has more effects on mitochondrial proteins than on cytosolic proteins. Ethanol oxidation can facilitate the production of superoxide by transferring an electron to oxygen [164,165,166]. Studies have suggested that mitochondria play a role in the oxidative stress response of the liver to alcohol [167]. Antioxidant therapy could be used to treat or prevent ALD [168]. Many natural products with abundant antioxidants are effective in preventing liver from oxidative stress. Bioactive compounds in plants, vegetables, and fruits are also involved in preventing oxidative damage in the liver [169].

### 4.2. Nonalcoholic Fatty Liver Disease

The most common chronic liver disease is nonalcoholic fatty liver disease (NAFLD) [170]. It has a major role in inducing oxidative stress. NAFLD is a result of disturbance in lipid metabolism, which in turn can accumulate fat, leading to NAFLD. This affects ROS generators such as mitochondria and ER. NADPH oxidase functions by inducing ROS in case of increased lipid accumulation. Mitochondria through the ETC can produce ROS. Non-ETC, such as fatty acid (FA) β-oxidation, can also produce ROS [171,172]. NADPH oxidase and endoplasmic stress related to NAFLD remain unclear. NAFLD is known to play an important role in the risk of end-stage liver disease. Diabetes and obesity are currently the most considered conditions where NAFLD has become a common chronic liver disease. Experiments on animals have shown that antioxidants can reduce liver damage by lipid accumulation [173,174]. Clinical trials have shown the effects of antioxidants on NAFLD [175]. The pathogenic spectrum of NAFLD includes insulin resistance, oxidative stress, and inflammation. Records suggest that those with insulin resistance are at a higher risk of having NAFLD [176]. Increased FA and other metabolites can induce oxidative phosphorylation, resulting in ROS and oxidative stress. Higher levels of lipid accumulation can lead to liver dysfunction and damage. Lipid peroxidation is initiated by polyunsaturated FA, which can increase ROS production and affect membrane permeability. Mitochondrial respiratory chain activity is reduced by mitochondria lipid peroxidation, which produces ROS and oxidative stress. Dietary supplements such as acai, vitamin E, and vitamin C as natural antioxidants have been studied using models to treat or prevent NAFLD. They can increase the GPx level and reduce ROS production and oxidative stress [176,177].

### 4.3. Acute Liver Disease

Acute liver disease is a common condition which is reversible. Acute hepatic damage can result in a rapid decline of liver function with hepatic encephalopathy occurring 26 weeks from the first symptoms of liver dysfunction [178]. Drug-induced liver injury, along with viral hepatitis, is a common cause of acute liver failure (ALF). Antibiotics are major causes of drug-induced liver injury, which can induce ALF, and neuropsychoactive drugs can also cause acute liver injury [179]. The pathophysiology of acute liver injury is divided into primary ALF and secondary multiorgan failure (MOF) [180]. In primary ALF, acetaminophen (APAP) is a nontoxic drug that is glucuronidated and sulfated by uridine 5′ diphospho-glucuronosyltransferase and sulfotransferase into nontoxic compounds that are excreted in the urine. Excess APAP is converted into a toxic compound by CYP450 enzyme, which is then conjugated with GSH to detoxify the toxic compound [181]. Drug overdose can result in GSH reduction and covalent binding to hepatocellular proteins and mitochondrial oxidative stress [182]. Secondary MOF is related to sepsis, an anti-inflammatory response syndrome caused by cytokines IL-4 and IL-10 [183,184]. Neutrophil has functions in damage caused by ROS production, reduced phagocytic response, and so on [185].

## 5. Future Aspects

Oxidative stress is a common process involved in various liver diseases. Gene alterations or modifications during oxidative stress are helpful for understanding and monitoring early liver disease conditions. To recognize liver damage or injury upon oxidative stress, post-transgenomic studies on molecular gene expression patterns are needed. Gene expression patterns can be studied by using ARE genes as biomarkers for treating and detecting liver damage and disease early. Each ARE candidate gene should be further studied to understand the role of oxidative stress in maintaining redox homeostasis. Since only a few candidate genes have been studied as of now, information on other antioxidant genes in response to oxidative stress is lacking. Another future aspect in measuring gene expression is by proteomic studies and DNA assays, since DNA and proteins are directly involved in maintaining the redox condition through signaling pathways during an oxidative stress. As most of the drugs mainly target proteins, adequate proteomics information regarding antioxidant candidates will help in various stages of effective drug discovery, such as analyzing novel targets, drug designing, and understanding the possible mechanism of action [186,187]. However, current proteomics knowledge of the ROS–antioxidant system is meager, as the majority of the studies are performed at the RNA level [188].

## 6. Conclusions

Oxidative stress is caused by an imbalance between free radicals and antioxidants involved in cell damage. Maintaining the redox condition during oxidative stress is a major concern to prevent the liver from damage or injury. Redox homeostasis is maintained by redox signaling pathways of the natural antioxidant system, which in turn can activate specific AREs. AREs are important for regulating stress response gene expression in the liver. Antioxidant target genes are regulated by master transcriptional regulators in the liver in response to oxidative stress. The downregulation of these target genes can result in excessive oxidative stress response. Identified oxidative/antioxidant genes can act as markers in the future, paving the way for the early detection of liver damage caused by oxidative stress.

## Data Availability

Not applicable.
